# Components of the sympathetic nervous system as targets to modulate inflammation – rheumatoid arthritis synovial fibroblasts as neuron-like cells?

**DOI:** 10.1186/s12950-023-00336-z

**Published:** 2023-03-14

**Authors:** Xinkun Cheng, Torsten Lowin, Nadine Honke, Georg Pongratz

**Affiliations:** 1 Clinic for Rheumatology & Hiller Research Center, Life Science Center, University Hospital Duesseldorf, Merowingerplatz 1A, 40225 Duesseldorf, Germany; 2 Department of Orthopedics, Nanjing BenQ Medical Center, The Affiliated BenQ Hospital of Nanjing Medical University, Nanjing, China; 3 Center for Rheumatologic Rehabilitation, Asklepios Hospital Bad Abbach, Medical Faculty of the University of Regensburg, 93077 Bad Abbach, Germany; 4 Medical Faculty of the University of Regensburg, 93053 Regensburg, Germany

**Keywords:** Rheumatoid arthritis, Synovial fibroblasts, Catecholamine, Dopamine, Norepinephrine, TNF, IL-6

## Abstract

**Background:**

Catecholamines are major neurotransmitters of the sympathetic nervous system (SNS) and they are of pivotal importance in regulating numerous physiological and pathological processes. Rheumatoid arthritis (RA) is influenced by the activity of the SNS and its neurotransmitters norepinephrine (NE) and dopamine (DA) and early sympathectomy alleviates experimental arthritis in mice. In contrast, late sympathectomy aggravates RA, since this procedure eliminates anti-inflammatory, tyrosine hydroxylase (TH) positive cells that appear in the course of RA. While it has been shown that B cells can take up, degrade and synthesize catecholamines it is still unclear whether this also applies to synovial fibroblasts, a mesenchymal cell that is actively engaged in propagating inflammation and cartilage destruction in RA. Therefore, this study aims to present a detailed description of the catecholamine pathway and its influence on human RA synovial fibroblasts (RASFs).

**Results:**

RASFs express all catecholamine-related targets including the synthesizing enzymes TH, DOPA decarboxylase, dopamine beta-hydroxylase, and phenylethanolamine N-methyltransferase. Furthermore, vesicular monoamine transporters 1/2 (VMAT1/2), dopamine transporter (DAT) and norepinephrine transporter (NET) were detected. RASFs are also able to degrade catecholamines as they express monoaminoxidase A and B (MAO-A/MAO-B) and catechol-O-methyltransferase (COMT). TNF upregulated VMAT2, MAO-B and NET levels in RASFs. DA, NE and epinephrine (EPI) were produced by RASFs and extracellular levels were augmented by either MAO, COMT, VMAT or DAT/NET inhibition but also by tumor necrosis factor (TNF) stimulation. While exogenous DA decreased interleukin-6 (IL-6) production and cell viability at the highest concentration (100 μM), NE above 1 μM increased IL-6 levels with a concomitant decrease in cell viability. MAO-A and MAO-B inhibition had differential effects on unstimulated and TNF treated RASFs. The MAO-A inhibitor clorgyline fostered IL-6 production in unstimulated but not TNF stimulated RASFs (10 nM-1 μM) while reducing IL-6 at 100 μM with a dose-dependent decrease in cell viability in both groups. The MAO-B inhibitor lazabemide hydrochloride did only modestly decrease cell viability at 100 μM while enhancing IL-6 production in unstimulated RASFs and decreasing IL-6 in TNF stimulated cells.

**Conclusions:**

RASFs possess a complete and functional catecholamine machinery whose function is altered under inflammatory conditions. Results from this study shed further light on the involvement of sympathetic neurotransmitters in RA pathology and might open therapeutic avenues to counteract inflammation with the MAO enzymes being key candidates.

**Supplementary Information:**

The online version contains supplementary material available at 10.1186/s12950-023-00336-z.

## Introduction

The sympathetic nervous system (SNS) is a comprehensive system mediating the ‘fight and flight’ response and is indispensable for body homeostasis during all kinds of activities [1]. It is a constant regulatory machinery transmitting signals from the central nervous system to target tissues thereby regulating their function [2].

The catecholamines norepinephrine (NE), epinephrine (EPI) and dopamine (DA) are the neurotransmitters of the SNS and are of pivotal importance in regulating inflammation and numerous physiological and pathological processes. In the central nervous system, abundant studies have illustrated the constitution and function of the catecholaminergic pathway. It is also clear, that immune cells have the capability to produce and sense catecholamines resulting in modulation of immune function [3].

Rheumatoid arthritis (RA), a chronic autoimmune disorder, is characterized by cartilage and bone damage accompanied by disability causing numerous clinical problems including persistent synovitis and articular dysfunction [4]. The SNS and its neurotransmitters play a dual role in disease onset and severity. In previous studies, it was shown that sympathectomy before the induction of experimental arthritis in mice reduces arthritic score and onset while late sympathectomy aggravated the disease. This suggests a switch from a pro- to an anti-inflammatory effect during the course of RA [5]. The anti-inflammatory effect might be due to newly appearing tyrosine hydroxylase (TH) positive catecholamine-producing cells which have been detected in synovial tissue of RA and osteoarthritis (OA) patients [6]. Mixed synovial cells were assumed to produce and release DA, representing a noncanonical mechanism in the modulation of local joint inflammation by inhibiting tumor necrosis factor (TNF) release [7]. Moreover, it was reported that RA synovial fibroblasts (SFs) possess a dopaminergic system, including dopamine receptors and their activation resulted in a reduction of inflammatory cytokine release by RASFs [8].

However, it is still unclear whether RASFs can produce and store NE or EPI and contribute to catecholamine degradation. Therefore, in this study we investigated the catecholaminergic pathway and its function in RASFs. With the MAO enzymes, we identified a potential therapeutic target, which might help to control cytokine release by RASFs.

## Results

### RASFs express all components to synthesize, transport, store, and degrade catecholamines – selective regulation by TNF

**Fig. 1:**
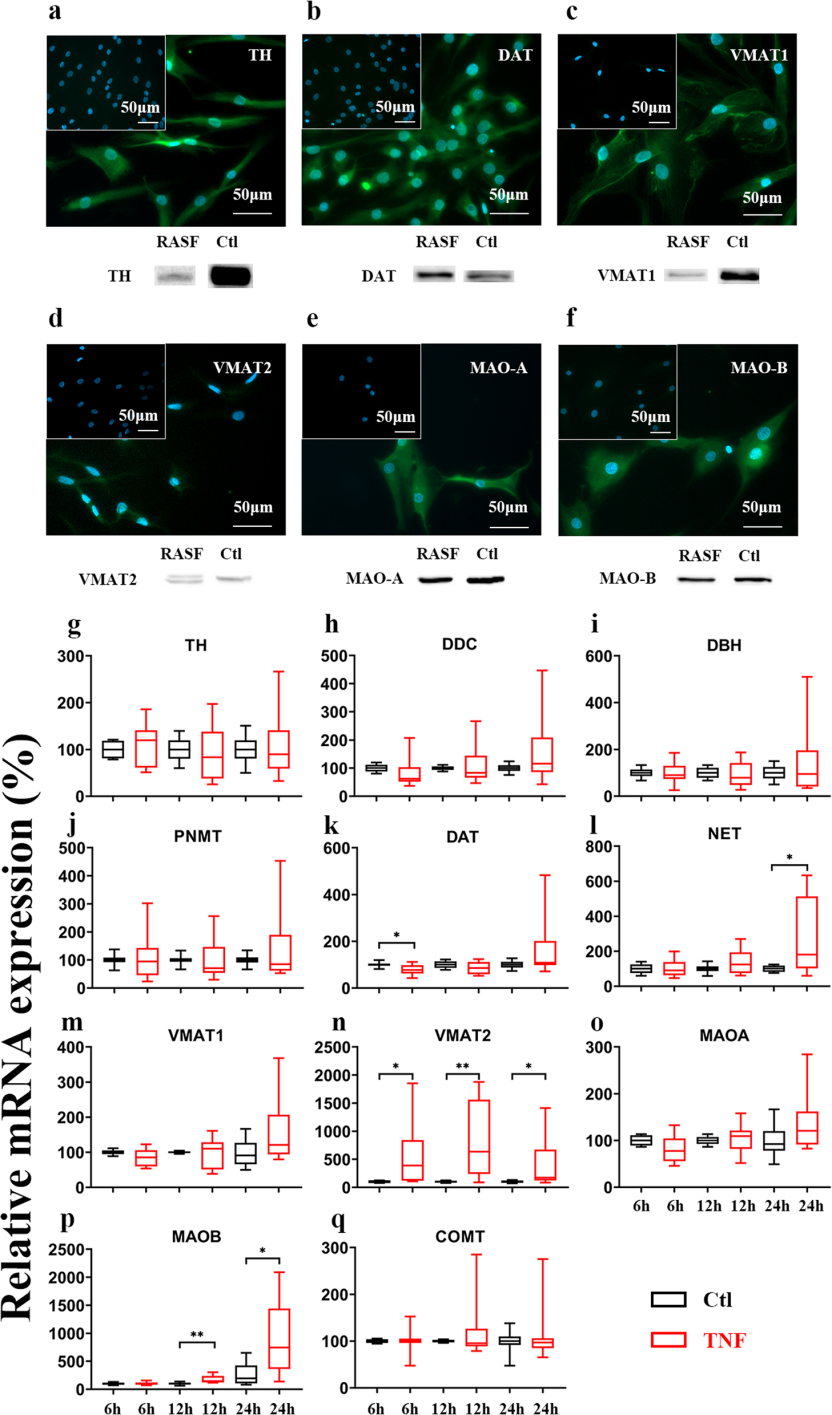
Expression of components of the catecholaminergic pathway in rheumatoid arthritis synovial fibroblasts (RASFs) under basal conditions and after stimulation with tumor necrosis factor (TNF). **a-f** Immunofluorescence (IF) and western blot (WB) images of dopamine transporter (DAT) (**a**), tyrosine hydroxylase (TH) (**b**), monoamine oxidase-A (MAO-A) (**c**), monoamine oxidase-B (MAO-B) (**d**), vesicular monoamine transporter 1 (VMAT1) **(e)** and vesicular monoamine transporter 2 (VMAT2) (**f**). Staining with respective antibody isotypes (upper left) served as negative control, target proteins are shown in green, cell nuclei (blue) were stained with DAPI (Bar 50 μm). Mouse brain homogenate was used as positive control (Ctl) in WB experiments. **g-q** Relative mRNA expression of components of the catecholaminergic pathway in RASFs with and without TNF stimulation for 6-, 12- and 24-hours including TH (**g**), DOPA decarboxylase (DDC) (**h**), dopamine-beta-hydroxylase (DBH) (**i**), phenylethanolamine N-methyltransferase (PNMT) (**j**), norepinephrine transporter (NET) (**k**), DAT (**l**), VMAT1 (**m**), VMAT2 (**n**), MAO-A (**o**), MAO-B (**p**) and Catechol-O-methyltransferase (COMT) (**q**). (*n* = 5) * *p* < 0.05, ** *p* < 0.01, comparing with the control group of each stimulation at a given time point by paired Student’s t-test. Black, control conditions; Red: stimulated with TNF (10 ng/mL); 6 h, 12 h, 24 h = stimulated time in hours

**Fig. 2:**
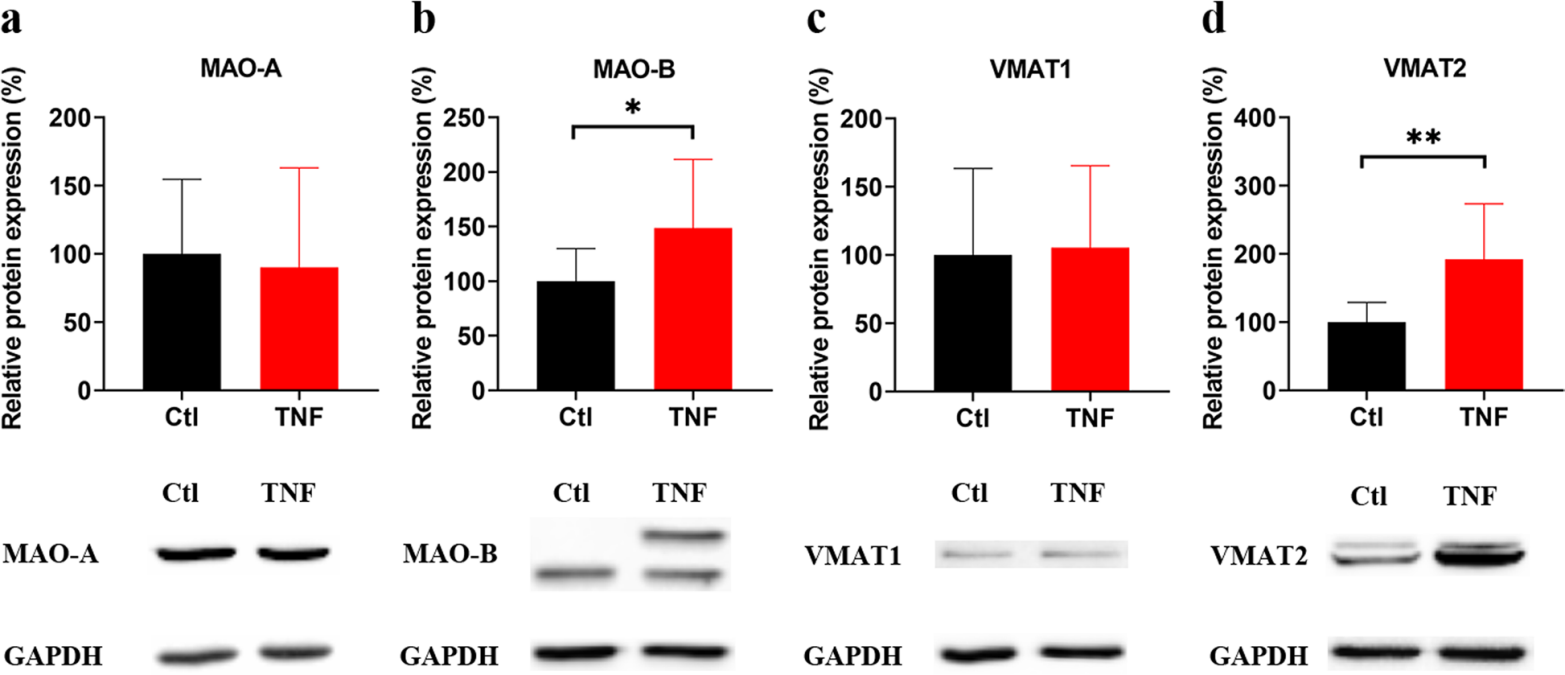
Relative protein expression of MAO-A, MAO-B, VMAT1 and VMAT2 after TNF stimulation. Synovial fibroblasts were treated with TNF (10 ng/mL) for 72 h or left untreated. Protein levels of MAO-A (**a**), MAO-B (**b**), VMAT1 (**c**) and VMAT2 (**d**) determined by WB. (*n* = 5) * *p* < 0.05, for comparisons between TNF treated and untreated cells by paired t-test. Ctl (black), control group without TNF stimulation; TNF (red), tumor necrosis factor stimulated

In a first step, we detected key proteins involved in the synthesis (tyrosine hydroxylase, TH), catecholamine uptake and storage (dopamine transporter, DAT; vesicular monoamine transporters 1 and 2, VMAT 1/2) and degradation (monoamine oxidases A and B, MAO-A/B) by Immunofluorescence (IF) and Western Blotting (WB) in RASF (Fig. 1a-f; and supplementary fig. 1). As some antibodies were not specific in western blotting, we also detected all target proteins by quantitative polymerase chain reaction (qPCR). In addition, we confirmed the expression of DOPA decarboxylase (DDC), dopamine-beta-hydroxylase (DBH), phenylethanolamine N-methyltransferase (PNMT), Catechol-O-methyltransferase (COMT) by qPCR (Fig. 1g). Since RASF are subjected to pro-inflammatory cytokines in the joint, we also investigated the regulation of these mRNAs at different time points under the influence of TNF. We found DAT to be reduced at 6 h (down 20.68 ± 7.825%, *p* = 0.0166) by TNF and NET (up 179.4 ± 68.20%, *p* = 0.0170) to be increased after 24 h, but the magnitude of regulation was small (Fig. 1g). However, VMAT2 (up 796.3 ± 217.3% at 12 h, *p* = 0.0018) and MAO-B (up 650.4 ± 229.2% at 24 h, *p* = 0.0109) were strongly upregulated by TNF treatment (Fig. 1g). Since VMAT and MAO were influenced by TNF on mRNA level, we also confirmed these results by WB (Fig. 2 and supplementary fig. 1). Here, we demonstrated that TNF selectively increases VMAT2 and MAO-B but not VMAT1 or MAO-A (Fig. 2b, d). Interestingly, TNF stimulation induces a specific isoform of MAO-B or introduces a posttranslational modification, as western blotting revealed the appearance of a second band with a slightly higher molecular weight (Fig. 2b).

### The catecholamine machinery in RASFs is functional

**Fig. 3:**
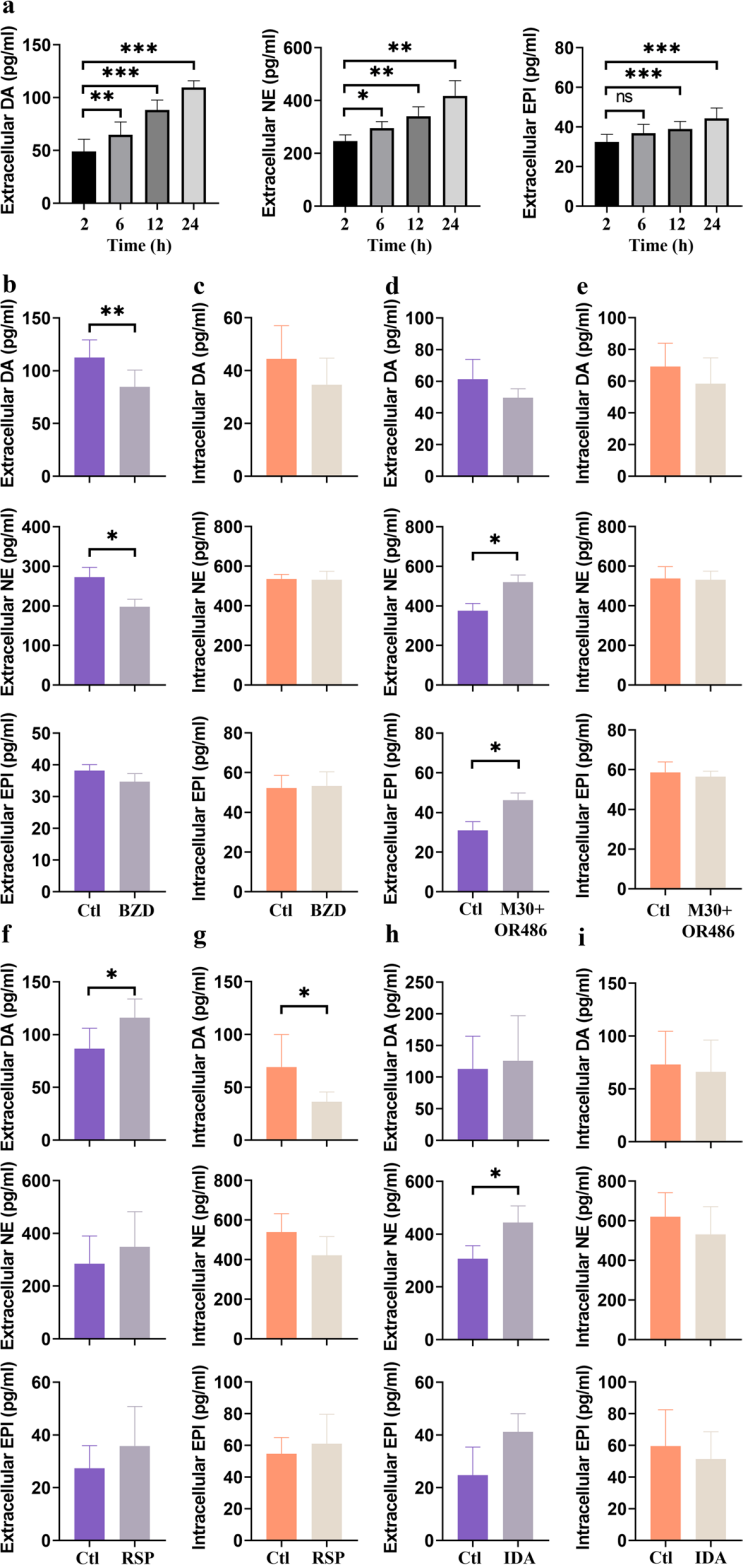
Catecholamine production by RASFs. **a** Time-dependent synthesis of dopamine (DA), norepinephrine (NE) and epinephrine (EPI) by RASF. Extracellular levels of catecholamines after 2-, 6-, 12- and 24-hours incubation with complete medium are shown. **b**, **c** Extracellular (**b**) and intracellular (**c**) catecholamine levels after 72-hour inhibition of DDC with benserazide hydrochloride (BZD, 50 μM). **d**, **e** Extracellular (**d**) and intracellular (**e**) catecholamine levels after 24-hours inhibition of MAO and COMT with M30 (MAO inhibitor, 10 μM) and OR486 (COMT inhibitor, 10 μM). **f**, **g** Extracellular (**f**) and intracellular (**g**) catecholamine levels after 24-hours inhibition of VMAT with reserpine (RSP, 10 μM). **h**, **i** Extracellular (**h**) and intracellular (**i**) catecholamine levels after 24-hours inhibition of DAT and NET with indatraline hydrochloride (IDA, inhibitor of both DAT and NET, 10 μM). (*n* = 4–5) * *p* < 0.05, ** *p* < 0.01, *** *p* < 0.001, for comparisons between inhibitor treated and untreated groups by MANN-Whitney U test, and for comparisons between 2-hour-incubation group and the other three incubation time groups by one-way ANOVA with Tukey’s post-hoc test

Since RASF have all necessary enzymes to synthesize DA, NE and EPI, we assessed whether these catecholamines are actually produced by these cells. As shown in Fig. 3, the concentration of extracellular catecholamines was increased continuously from 2 hours to 24 hours. The strongest increase was found with DA, as levels at baseline were around 49 pg/ml (0.33 nM) but gradually increased over time reaching around 110 pg/mL (0.72 nM) after 24 h (+ 60.54 ± 13.17 pg/mL, *p* = 0.0002, Fig. 3a). A similar increase was found for NE, whose levels also increased from 246 pg/mL (1.46 nM) at 2 hours to 417 pg/mL after 24 h (2.47 nM; + 171.2 ± 61.88 pg/mL, *p* = 0.0077, Fig. 3a). Extracellular EPI was also increased from 32 pg/mL (0.17 nM) to 44 pg/mL (0.24 nM) after 24 h (+ 11.93 ± 6.53 pg/mL; p = 0.0002) but overall levels were much lower compared with DA or NE (Fig. 3a). When the conversion from L-DOPA to DA was blocked by the DDC inhibitor benserazide hydrochloride (BZD, 50 μM), both extracellular DA (p = 0.007) and NE (*p* = 0.038) levels declined significantly (Fig. 3b). A similar trend was observed for EPI (Fig. 3b). The intracellular catecholamine content was not altered by treatment with BZD (Fig. 3c).

We also investigated whether inhibition of other components of the catecholaminergic pathway alter levels of DA, NE and EPI. When MAO and COMT were inhibited by the combination of M30 (MAO inhibitor, 10 μM) and OR486 (COMT inhibitor, 10 μM), extracellular NE (*p* = 0.028) and EPI (p = 0.028) levels were increased (Fig. 3d), while extracellular DA and intracellular levels remained unchanged (Fig. 3d, e). Similarly, when catecholamine storage was targeted by the VMAT inhibitor and releaser reserpine (RSP, 10 μM), extracellular DA (*p* = 0.010) and intracellular DA (*p* = 0.032) was increased (Fig. 3f, g), while concomitantly, extracellular NE and EPI showed a decreasing trend (Fig. 3f, g). Targeting re-uptake of catecholamines with indatraline hydrochloride (IDA, 10 μM), an inhibitor of DAT and NET, increased extracellular levels of NE (*p* = 0.0317, Fig. 3h) while concomitantly extracellular levels of DA and EPI tended to decrease. No difference was found in intracellular levels of DA, NE and EPI (Fig. 3i).

### MAO-A, but not MAO-B inhibition increases extracellular catecholamine levels

**Fig. 4:**
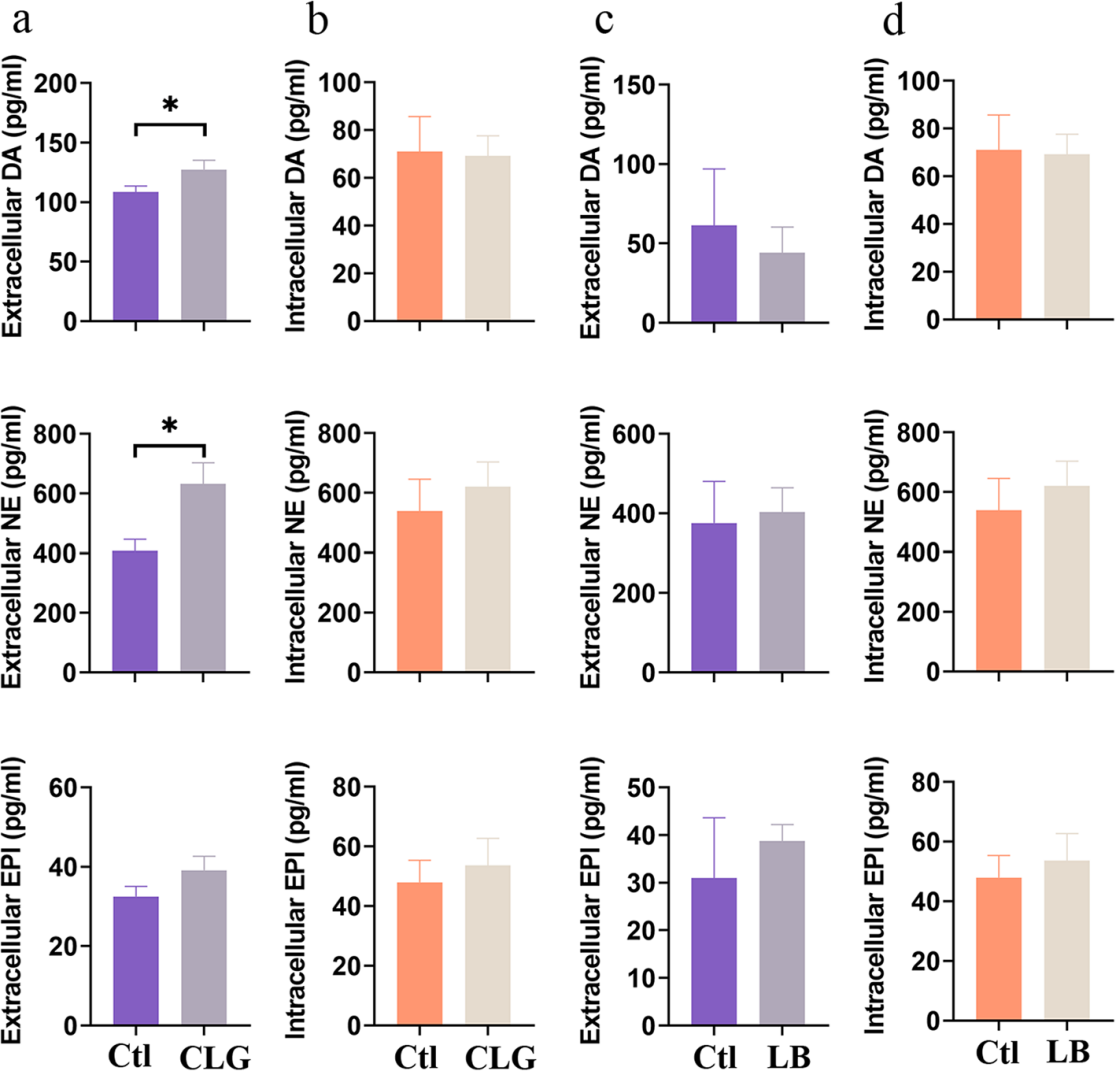
Extracellular and intracellular catecholamine levels after selective MAO-A or MAO-B inhibition. **a**, **b** Extracellular (**a**) and intracellular (**b**) catecholamine levels after 24-hour inhibition of MAO-A with clorgyline (CLG, 1 μM). **c**, **d** Extracellular (**c**) and intracellular (**d**) catecholamine levels after 24-hour inhibition of MAO-B with lazabemide hydrochloride (LB, 10 μM). (*n* = 4–6) * *p* < 0.05, for comparisons between inhibitor treated and untreated groups by MANN-Whitney U test

Since both MAO isoforms are able to degrade catecholamines, we used selective inhibitors to pinpoint the enzyme involved. After 24 hours stimulation, CLG (MAO-A inhibitor) significantly increased extracellular catecholamines (*p* = 0.0449 for DA and *p* = 0.0335 for NE, Fig. 4a), except for EPI, while the intracellular catecholamine levels remained unchanged (Fig. 4b). When using the selective MAO-B inhibitor LB, there was no significant modulation of intra- or extracellular catecholamine levels (Fig. 4c, d, *p* > 0.05).

### Exogenous DA and NE modulate interleukin 6 (IL-6) production and cell viability of RASFs

**Fig. 5:**
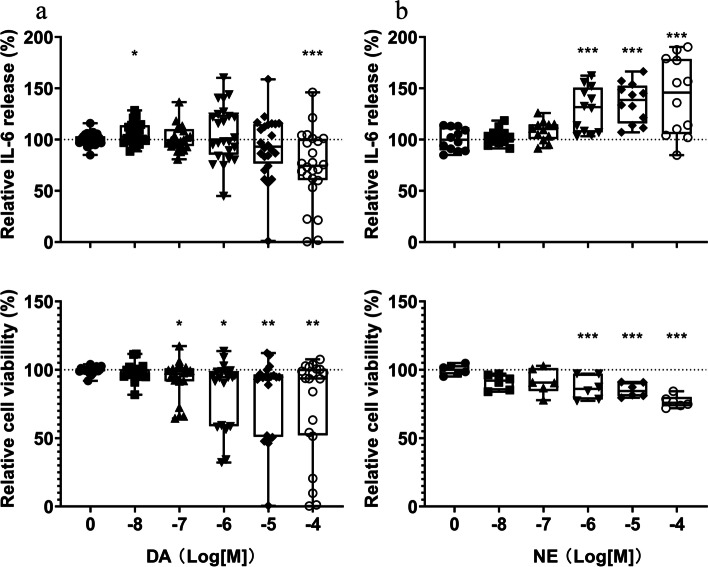
Dopamine and norepinephrine modulate interleukin (IL)-6 production and cell viability of RASFs. IL-6 production (**a**, upper panel) and cell viability (**a**, lower panel) after stimulation with dopamine (DA, 10 nM – 100 μM) for 24 hours (*n* = 6). IL-6 production (**b**, upper panel) and cell viability (**b**, lower panel) after stimulation with norepinephrine (NE, 10 nM – 100 μM) for 24 hours. (*n* = 3). * *p* < 0.05, ** *p* < 0.01, *** *p* < 0.001, for comparisons with the control group (without DA or NE) by one-way ANOVA with Tukey’s post-hoc test

Since RASFs are able to produce NE and DA, we were also interested whether these catecholamines regulate cell viability and the production of IL-6, a major cytokine produced by RASFs. After stimulation over 24 hours, we found DA at 100 μM to slightly decrease IL-6 production (Fig. 5a, upper panel), whereas cell viability was reduced already at concentrations above 100 nM (Fig. 5a, lower panel). In contrast, NE increased IL-6 production in concentrations greater than 1 μM reaching a maximum at 100 μM (+ 40%, *p* < 0.0001, Fig. 5b, upper panel). However, this was also accompanied by a dose-dependent reduction of cell viability starting at 1 μM with a maximal decrease of 24% at 100 μM (*p* < 0.0001, Fig. 5b, lower panel).

### Modulation of catecholamine synthesis by TNF

**Fig. 6:**
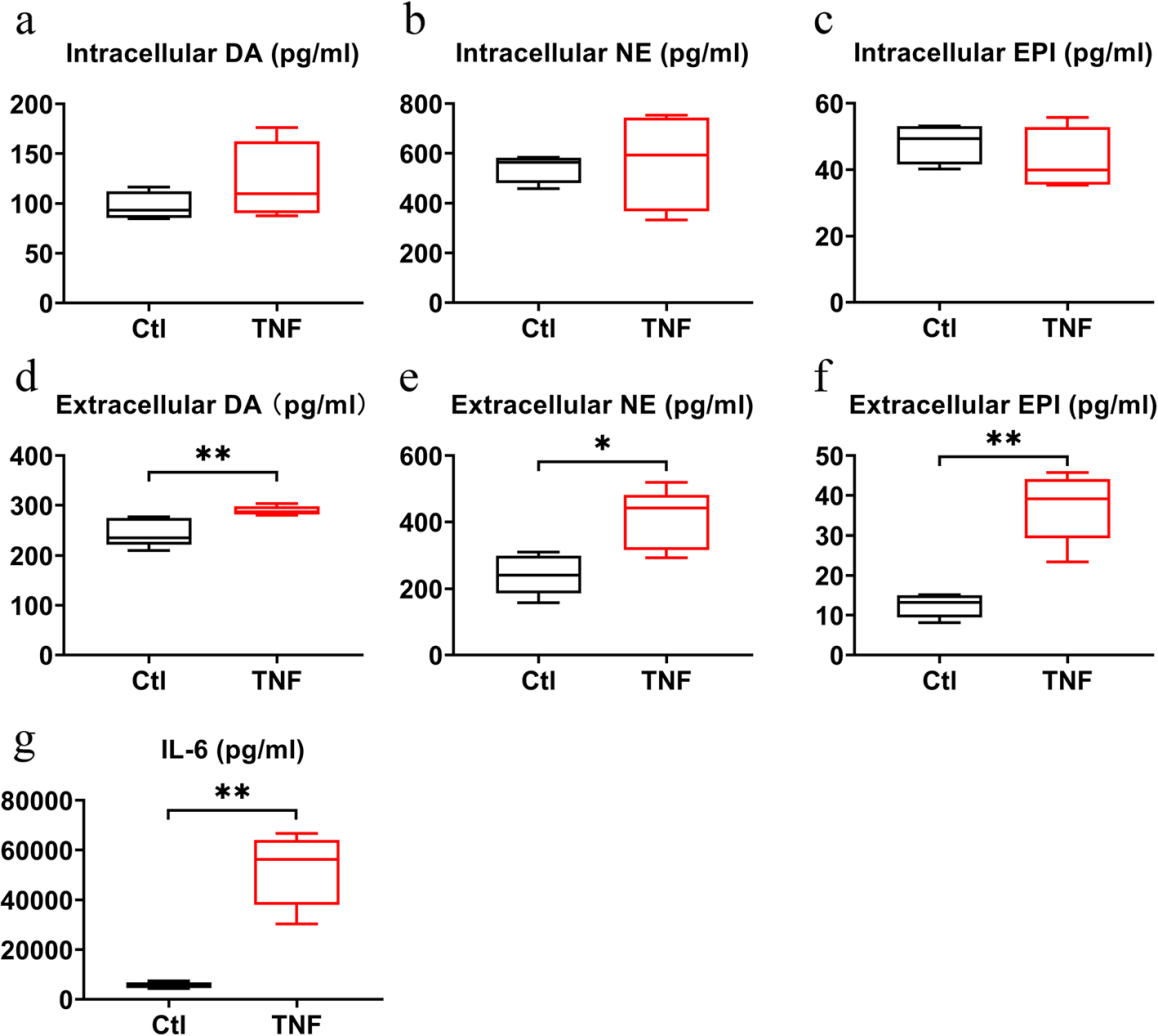
Modulation of intra- and extracellular catecholamine levels and IL-6 production by TNF. **a-f** Intracellular (**a-c**) and extracellular (**d-f**) levels of dopamine (DA) (**a**, **d**), norepinephrine (NE) (**b**, **e**) and epinephrine (EPI) (**c**, **f**) levels under basal conditions and after stimulation with TNF (10 ng/mL for 24 h. (*n* = 4–5) **g** IL-6 production in 24 h after stimulation with TNF vs unstimulated controls (*n* = 3). * *p* < 0.05, ** *p* < 0.01, for comparisons of catecholamine levels versus control by unpaired t-test in a-f, for the comparison of IL-6 production versus control MANN-Whitney U test was employed. Ctl (black), control group; TNF (red), tumor necrosis factor

Since RA is characterized by an inflammatory environment, we also evaluated catecholamine production under the influence of TNF. After 72 hours of stimulation with TNF [10 ng/ml], no obvious difference was found in intracellular catecholamine levels (Fig. 6a-c). However, extracellular DA (+ 18%), NE (+ 55%) and EPI (+ 208%) were significantly increased by TNF treatment (*p* < 0.05, Fig. 6d-f). This increase in catecholamines was accompanied by enhanced production of IL-6 (+ 890%, *p* = 0.0022, Fig. 6g).

### Impact of selective MAO-A or MAO-B inhibition on basal and TNF-induced IL-6 production and cell viability

**Fig. 7:**
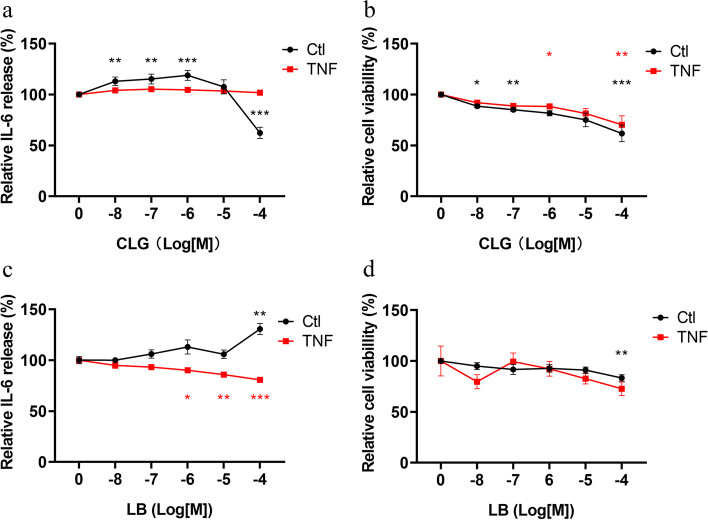
Modulation of IL-6 production and cell viability by selective MAO inhibition. IL-6 production and cell viability of RASFs treated with different concentrations of the MAO-A inhibitor clorgyline (CLG, *n* = 6) (**a**, **b**) or the MAO-B inhibitor lazabemide hydrochloride (LB, *n* = 5) (**c**, **d**). RASFs were pre-treated with TNF for 72 hours (red) or left untreated (black). MAO-A and MAO-B inhibitors were applied thereafter for 24 hours. * *p* < 0.05, ** *p* < 0.01, *** *p* < 0.001, comparing with the control group without MAOs inhibitors by one-way ANOVA with Tukey’s multiple comparison test

We found MAO-B to be highly upregulated by TNF (Figs. 1g and 2b), and, therefore we investigated whether inactivation of this enzyme with the selective MAO-B inhibitor (LB) has any impact on cell viability and IL-6 production by RASF. In addition, we also employed the selective MAO-A inhibitor (CLG) for comparison. When MAO-A was inhibited in RASFs without TNF pretreatment, IL-6 levels were elevated (+ 19% at 1 μM) by low concentrations of CLG (10 nM to 1 μM; p < 0.05) and declined at 100 μM CLG (− 38%; *p* < 0.0001, Fig. 7a). In contrast, there was no significant regulation of IL-6 by CLG in RASFs with TNF pretreatment (Fig. 7a). However, cell viability of RASFs was dose-dependently inhibited by CLG regardless of TNF stimulation (*p* < 0.05, Fig. 7b). MAO-B inhibition resulted in the opposite: LB significantly suppressed IL-6 production by TNF stimulated RASFs at concentrations above 1 μM with a maximum at 100 μM (− 19%, *p* < 0.0001, Fig. 7c). Without TNF pretreatment, LB increased IL-6 levels at 100 μM (+ 31%, *p* = 0.0038, Fig. 7c). Cell viability was slightly decreased by 100 μM LB in the control but not in the TNF pre-stimulated group (*p* < 0.01, Fig. 7d).

## Discussion

To our knowledge, this is the first study to comprehensively characterize the significance of the catecholaminergic machinery in RASF. We demonstrated that RASF not only take up and store catecholamines but are also able to degrade and synthesize DA, NE and EPI. In addition, we have shown that an inflammatory environment mimicked by the addition of TNF influences catecholamine levels but also MAO-B expression which in turn modulated IL-6 production.

Over the past decades, the pivotal influence of the SNS on regulating inflammation in RA was increasingly recognized [1]. Early reports already inferred that sympathectomy might be beneficial in the treatment of RA [9] and since then many other studies confirmed extensive crosstalk of the SNS with the immune system [10–13]. In fact, it has been shown that experimental arthritis is attenuated when β_2_-adrenergic receptor signaling is activated [14, 15]. Similarly, dopamine also influences RA pathology, as inhibition of D1-like or activation of D2-like dopamine receptors ameliorated collagen-induced arthritis in mice [16, 17]. Although the SNS aggravates experimental arthritis at an early stage [18], its effects are reversed during the course of the disease. SNS fibre density decreases in the joint, likely to nerve fibre repulsion initiated by semaphorin 3C production of macrophages and RASF [19, 20]. This decrease in SNS fibres is accompanied by the appearance of anti-inflammatory, TH positive cells in synovial tissue which are able to produce catecholamines [6, 7]. Although RASF have been identified as being TH positive [7], it was unclear until now whether these cells actively synthesize, store or degrade catecholamines. We found that RASF express all enzymes necessary (TH, DDC, DBH, PNMT) to catalyze the steps from tyrosine to epinephrine and, accordingly, we detected DA, NE and EPI both intra- and extracellularly. Although these enzymes were not regulated by TNF, we found increased production of catecholamines in response to this cytokine. TNF increases TH expression in monocytes [21] and our findings confirm results from Miller et al. who showed that increased inflammation is associated with higher TH immunoreactivity and NE production in synovial tissue [22]. In RASF, TNF might enhance catecholamine synthesis not only by increasing TH expression but also by increasing cofactors important for the activity of TH such as tetrahydrobiopterin (BH4) as demonstrated in glioma cells [23, 24]. TNF does increase tetrahydrobiopterin levels by augmenting the expression of BH4-synthesizing enzymes guanosine triphosphate cyclohydrolase I, 6-pyruvoyl tetrahydropterin synthase and sepiapterin reductase [25].

We assessed the effects of exogenous DA and NE on IL-6 production and cell viability of RASFs and found that DA reduced IL-6 levels and cell viability, whereas NE increased IL-6 while concomitantly decreasing cell viability. In a previous study, we found reduced IL-6 and IL-8 production by RASF when challenged with intermediate levels of DA, however culture conditions and incubation time were different compared to this study [8]. The increase of IL-6 by NE has already been briefly described [26] and the involvement of β adrenergic receptors in RA pathology is well established [14, 27, 28]. The reduction of cell viability by the beta-adrenergic agonists isoprenaline and salbutamol but not NE has been shown [27] and intracellular DA is associated with the generation of reactive oxygen species (ROS) and subsequent cell death in neuronal cells [29]. Since relatively high levels of NE and DA are necessary to elicit appreciable effects on cytokine production, it is likely that these catecholamines only engage in autocrine and paracrine signaling.

Besides synthesizing enzymes, RASF also express VMAT1 and VMAT2 which regulate catecholamine storage [30]. However, only VMAT2 was strongly upregulated by TNF which might indicate that, under inflammatory conditions, vesicle composition is altered. It has been shown that the affinity towards monoamines is 3-fold higher for VMAT2 which, in contrast to VMAT1, also transports histamine [30]. The increase in VMAT2 might come as a consequence of elevated catecholamine synthesis induced by TNF as it has been shown that high extracellular levels of catecholamines due to stress or drug intake upregulated VMAT2 levels [31, 32]. Interestingly, in western blot analyses we found two bands of VMAT2 with slightly different molecular weights. As VMAT2 localization and function is governed by glycosylation, phosphorylation and nitrosylation [33], TNF might not only regulate overall levels but also VMAT2 activity. Inhibition of VMAT with reserpine disrupts catecholamine storage and we found an increase of extracellular catecholamines. However, reserpine releases catecholamines intracellularly and thereby depletes monoamines as they are degraded by MAO and COMT enzymes [34]. The observed increase in extracellular catecholamines induced by reserpine might be dependent on negative regulation of DAT and NET function [35, 36].

DAT and NET were both found to be expressed by RASF and their ligation with the non-selective reuptake inhibitor indatraline increased extracellular DA, NE and EPI while depleting their levels intracellularly. This is in line with results obtained in healthy volunteers that received intravenous nomifensine, another inhibitor of NET and DAT with similar pharmacologic properties [37].

We recognize that RASF synthesize, store and take up catecholamines, but do they also engage in their degradation? This is clearly the case as we detected both MAO isoforms along with COMT and their combined inhibition elevated extracellular catecholamine levels. This confirms in vivo and in vitro effects of the COMT inhibitor OR-486 or non- selective MAO inhibitors as used in this study [38, 39]. Analogous to VMAT2, MAO-B was strongly upregulated by TNF on mRNA and protein level and this pro-inflammatory cytokine might also induce posttranslational modifications in MAO-B, which was at least indicated by western blot analyses. Although this hasn’t been investigated in previous studies, purification of recombinant MAO-B from yeast revealed extensive acetylation [40] and ubiquitination is required for its insertion into mitochondrial membranes [41]. We further delineated the effects of MAO-A and MAO-B inhibition and investigated IL-6 production and cell viability of RASF under basal and TNF-stimulated conditions. MAO-A inhibition without TNF pre-treatment increased IL-6 production by RASF, whereas TNF stimulation abrogated this effect and cell viability was reduced regardless of TNF stimulation. While there is no data available regarding the influence of MAO-A on RASF function, studies from macrophages suggest that MAO-A breaks down monoamines and by doing so, increases the production of ROS. As a consequence, anti-inflammatory M2 polarization is favored and MAO-A inhibition reversed this effect [42]. In line with this, we also observed an increase in IL-6 upon MAO-A inhibition and the decreased cell viability might be due to the reduced production of ROS at high concentrations of MAO-A inhibitor. Although ROS induces cell death in high concentrations, low to intermediate concentrations actually promote cell survival and proliferation [43, 44]. Consequently, ROS levels need to be strictly controlled as too much or too little might negatively affect cell survival. MAO-B was upregulated by TNF and its inhibition decreased IL-6 production but enhanced it in TNF-naïve RASFs while cell viability was only slightly reduced by high concentration of MAO-B inhibitor in the TNF-naïve group. Although in vitro, both MAO isoforms are able to degrade catecholamines, in vivo, MAO-A was found to be mainly responsible for NE and DA degradation [45, 46], whereas MAO-B is associated with γ-aminobutyric acid (GABA) synthesis and the generation of ROS [46–48]. Accordingly, we also observed an increase in catecholamine levels with MAO-A but not MAO-B inhibition. Our results are in line with those from Won et al. who showed anti-inflammatory effects of MAO-B inhibition on cytokine production and experimental arthritis in mice [48]. Since peripheral GABA is mainly anti-inflammatory [49], the major effect of MAO-B inhibition seems to rely on the reduction of ROS. TNF induces ROS and this is associated with activation of nuclear factor ‘kappa-light-chain-enhancer’ of activated B-cells (NFκB) with pro-inflammatory consequences [50, 51]. Therefore, reduction of ROS by MAO-B inhibition might reduce the activity not only of NFκB signaling but also of other pro-inflammatory ROS-dependent pathways such as Jun activated kinase (JNK) [52] or mitogen-activated kinase [53].

## Conclusions

**Fig. 8:**
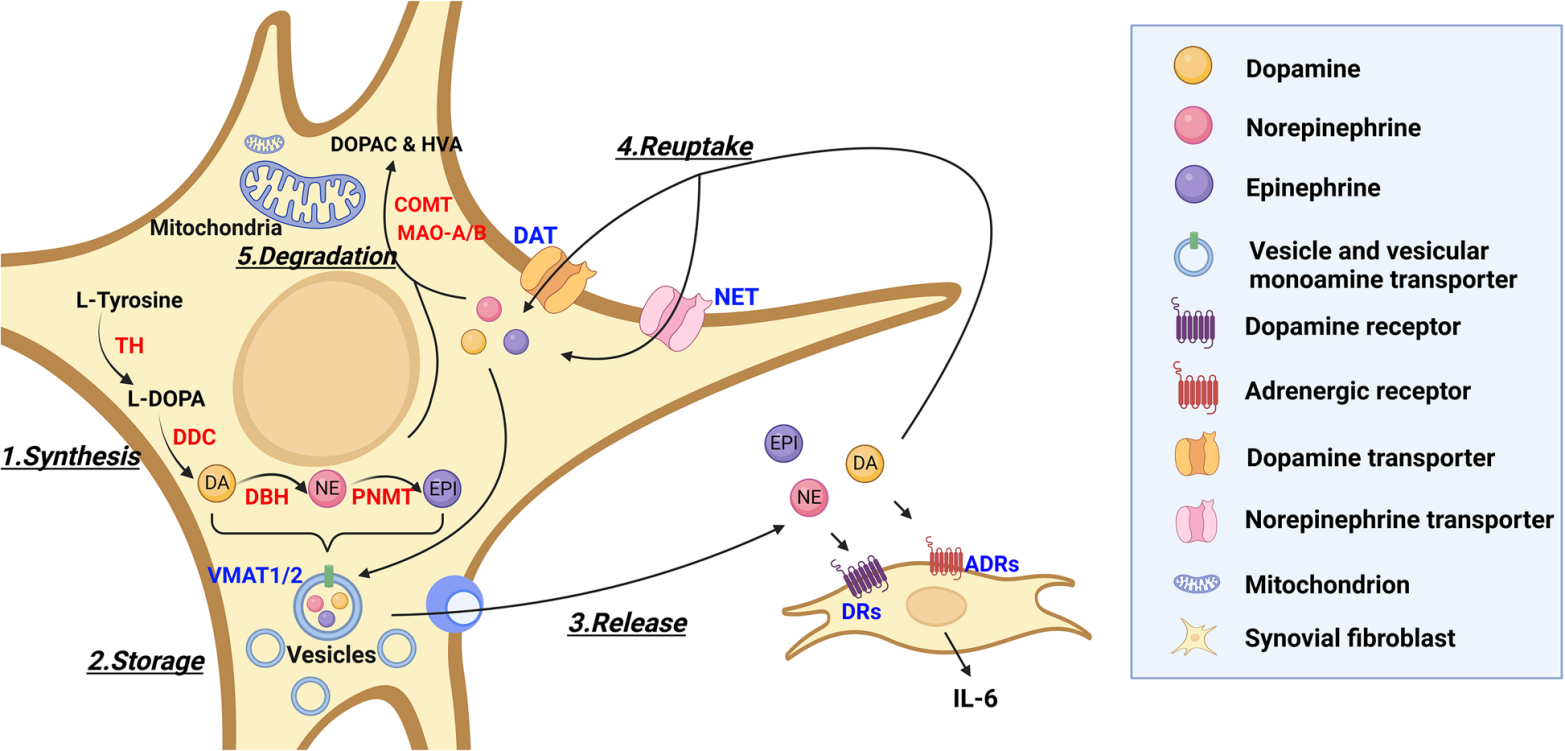
Schematic representation of the catecholaminergic pathway in RASFs. 1. Synthesis: RASF are able to synthesize DA, NE and EPI. This needs the participation of the enzymes TH, DDC, DBH and PNMT. 2. Storage: Vesicles are able to store catecholamines by transport through VMAT1 and VMAT2. 3. Release: Catecholamines can be released to the extracellular space by exocytosis and invoke downstream reactions via DRs and ADRs in an autocrine and paracrine fashion. Released catecholamines are able to ligate receptors (ADRs and DRs) and foster or inhibit secretion of IL-6. 4. Reuptake: Exogenous catecholamines are taken up through DAT or NET and repacked into vesicles or are degraded. 5. Degradation: MAO-A, MAO-B and COMT catalyze the degradation of excess catecholamines and thereby produce the metabolites DOPAC and HVA. (DA, dopamine; NE, norepinephrine; EPI, epinephrine; TH, tyrosine hydroxylase; DDC, DOPA decarboxylase; DBH, dopamine-beta-hydroxylase; PNMT, phenylethanolamine N-methyltransferase; VMAT1/2, vesicular monoamine transporter 1/2; DRs, dopamine receptors; ADRs, adrenergic receptors; DAT, dopamine transporter; NET, norepinephrine transporter; MAO-A/B, monoamine oxidase-A/B; COMT, catechol-O-methyltransferase; DOPAC, dihydroxy-phenyl acetic acid; HVA, homovanillic acid; IL-6, interleukin-6)

This is the first comprehensive study of the catecholaminergic system in RASF. We identified all components necessary for the production, storage, reuptake and degradation of catecholamines and an overview is depicted in Fig. 8. Therefore, RASFs, being of mesenchymal origin, resemble pre-synaptic sympathetic neuron-like cells in the joint. Similar to sympathetic neuron-associated macrophages, RASF might take up excess catecholamines from sympathetic nerves in the joint and store them for further use or participate in their degradation [54]. However, in situations where local catecholamine levels drop, e.g., after sympathetic nerve fibre repulsion during the course of arthritis, they might contribute to de novo catecholamine synthesis together with TH-positive lymphocytes and macrophages.

MAO inhibition might be an attractive therapeutic approach to target excess inflammation in RA. Very early studies already noted an improvement of RA under MAO inhibition [55] and MAO-B inhibition alone provided robust anti-inflammatory effects in experimental arthritis [48]. However, MAO-A also contributes to oxidative stress due to the degradation of catecholamines and it might be necessary to inactivate both isoforms (peripherally) for maximal effect on joint pathology. Therefore, MAO inhibitors might be investigated as adjunct therapy for RA.

## Methods

### Patients and compounds

In this study, 17 patients with long-standing RA fulfilling the American College of Rheumatology revised criteria for RA [56], who underwent elective knee joint replacement surgery, were included. Mean age was 70.12 ± 7.89 years for RA. Mean C-reactive protein (CRP) was 8.41 ± 11.94 mg/L for RA. Rheumatoid factor was 61.24 ± 69.57 IU/mL in RA. In the RA patient group 4/17 received methotrexate, 5/17 glucocorticoids and 5/17 received biologicals or Janus kinase inhibitors. All patients in this study were informed about the purpose and gave written consent before surgery. This study was approved by the Ethics Committees of the University of Düsseldorf (approval number 2018–87-KFogU). The compounds and chemicals with abbreviation, order number, company and concentration used are presented in Table 1.

**Table 1:** Compounds and chemicals used in this study

Compound	Abbreviation	Order number	Company	Concentration
Tumor necrosis factor	TNF	300-01A	PeroTech	10 ng/ml
Benserazide hydrochloride	BZD	B7283	Sigma-Aldrich	50 μM
Clorgyline	CLG	M3778	Sigma-Aldrich	1 μM
Reserpine	RSP	2742	Tocris / Bio-Techne	10 μM
M30 dihydrochloride	M30	6067	Tocris / Bio-Techne	10 μM
Lazabemide hydrochloride	LB	2460	Tocris / Bio-Techne	10 μM
OR-486	OR-486	0483	Tocris / Bio-Techne	10 μM
Indatraline hydrochloride	IDA	1588	R&D / Biotechne	10 μM

### Synovial tissue preparation and SFs culture

The RASF isolation and preparation was performed as described previously for in-vitro experiments [57]. Briefly, synovial tissue samples were immediately collected (up to 9 cm^2^) upon exposing knee joint capsule. The tissue pieces were carefully cut up into tiny fragments and digested with liberase (Roche Diagnostics, Mannheim, Germany) overnight at 37 °C. The filtration (70 μm) and centrifugation (300 g, 10 min) of the resulting suspension were carried out subsequently. After that, the pellet was obtained, which was then treated with erythrolysis buffer (20.7 g NH_4_Cl, 1.97 g NH_4_HCO_3_, 0.09 g EDTA and 1 L H_2_O) for 5 minutes. The suspension was centrifuged again for 10 minutes at 300 g. At last, RASFs were resuspended in RPMI-1640 (sigma Aldrich, St. Louis, USA) with 10% FCS. After culturing overnight, cells were treated with fresh medium to wash off dead cells and debris.

### WB

Cells were collected for the isolation of protein using RIPA lysis buffer (R 0278; Sigma) with complete protease inhibitor (Roche, Mannheim, Germany). Protein was quantified and subjected to electrophoresis using 12.5% SDS-polyacrylamide gels with the same amount of total protein, running for 60 min at 20 mA (Biorad, Puchheim, Germany). The gels were transferred onto a nitrocellulose membrane (Biorad) at 300 mA for 90 min. Then, membranes were blocked for 1 hour with 5% no-fat milk in TBS-T (Tris-Glycine-SDS Buffer from Sigma containing 0.1% Tween 20) at room temperature. The following primary antibodies shown in Table 2 were applied for overnight incubation at 4 °C. These antibodies are applicable to both human and mouse samples. Subsequently, the membranes were incubated with secondary antibody (goat anti-rabbit IgG HRP, DAKO P0448, 1:2000 in 5% no/fat milk) for 2 hours. Immunoreactive protein bands were visualized by ECL Prime (GE Healthcare, Freiburg, Germany). Membranes were washed three times with TBS-T for 5 min between each step. Proteins of interest were quantified in a V3 Western Workflow (Biorad) and the signals were normalized against that of GAPDH.

**Table 2:** Primary antibodies used in this study. WB = western blotting, IF = Immunofluorescence

	Catalog	Company	Dilution in WB	Dilution in IF
DAT	22,524–1-AP	Proteintech	1:1000	1:250
TH	25,859–1-AP	Proteintech	1:10000	1:500
VMAT1	ATM-007	Alomone	1:200	1:100
VMAT2	ATM-006	Alomone	1:400	1:1000
MAOA	10,539–1-AP	Proteintech	1:3000	1:300
MAOB	12,602–1-AP	Proteintech	1:4000	1:50
GAPDH	2118	CellSignalling	1:2000	–

### Immunofluorescence (IF)

IF was performed on cultured RASFs seeded in 96-well plates at approximately 80–90% confluence. Cells were washed with PBS 5 times and fixed with cold methanol for 20 min at − 20 °C. After rinsing 4 times with PBS, cells were permeabilized with 0.3% (v/v) Triton X-100 diluted in PBS for 5 min at room temperature. Afterwards, cells were blocked for 1 h at room temperature in blocking buffer (PBS with 5% normal swine serum (NSS, Dako, X0901)). Primary antibodies (Table 2) and the same amount of rabbit IgG polyclonal isotype (ab37415, abcam) were separately diluted in blocking buffer. After overnight incubation with primary antibodies or the IgG isotype at 4 °C, cells were washed with 0.3% (v/v) Triton X-100 diluted in PBS 4 times followed by 2 washes with PBS only. Then, secondary antibody (Goat anti-rabbit IgG (Alexa Fluor 488), ab150077, Abcam) labeled with Alexa Fluor 488-FITC was added in blocking buffer for 1 hour at room temperature. At last, cells were rinsed 6 times and covered with the ProLong™ Gold Antifade Mountant with DAPI (P36931, Invitrogen) and coverslips were kept in the dark overnight at 4 °C. Images of each target were captured by using an Axio Observer microscope (Zeiss-Germany) with a digital camera AxioCam (Zeiss-Germany). Zen 2.6 software (Blue edition-Zeiss-Germany) was used to analyze the images.

### Catecholamine enzyme-linked immunosorbent assay (ELISA)

For the determination of extracellular catecholamine, cells seeded in 96-well plates were cultured until confluence reached 90%. Supernatants were discarded, fresh RPMI with 10% FCS (50 μl/well) with specific inhibitors was added. The supernatants were collected according given protocols (E-EL-0045, E-EL-0046, E-EL-0047, Elabscience). Supernatants were collected after 2, 6, 12 and 24 hours and quantified.

To obtain intracellular catecholamines, cell lysates were prepared according to the manufacturer’s instructions. Briefly, cells were seeded in 6-well plates and incubated until at least 90% confluence. After stimulation, cells were collected and centrifuged at 300 g. The cell pellets were subsequently washed with pre-cooled PBS and suspended in distilled water. Cell lysis was achieved by repeated freeze-thaw cycles and by using an ultrasonic cell disrupter. Supernatants were collected after centrifugation and were used in ELISA.

### qPCR

Cultured cells with and without TNF treated were harvested after 6, 12 and 24 hours. Total RNA was extracted with the RNA Mini Kit (Qiagen, Hilden, Germany) according to manufacturer’s instructions. By spectrophotometry (260 nm), the content of total RNA was measured. Afterwards, the synthesis of cDNA was performed with iScript™ gDNA clear cDNA Synthesis Kit (BIO-RAD) from the same amount of RNA (1 μg) of different samples. qPCR was performed using qPCRBIO SyGreen Mix Hi-ROX (PCR Biosystems) in a total volume of 20 μl and the StepOnePlus real-time PCR system with the primers shown in Table 3. GAPDH was used as a quantitative control for normalization. The relative expression fold-change was expressed by the values of 2^-ΔΔCT^ [58]. Each qPCR analysis was conducted at least in duplicates.

**Table 3:** Primer sequences used in this study

Genes	Primer sequences	
	Forward	Reverse
TH	5′-TGTCCACGCTGTACTGGTTC-3′	5′-AGCTCCTGAGCTTGTCCTTG-3′
DDC	5′-GAACAGACTTAACGGGAGCCTTT-3′	5′-AATGCCGGTAGTCAGTGATAAGC-3′
DBH	5′-GACGCCTGGAGTGACCAGAA-3′	5′-CAGTGACCGGAACGGCTC-3′

### Il-6 ELISA

RASFs (10,000 cells/well) were seeded in 96-well plates at least 24 hours before stimulation. Supernatants were collected after indicated time for the quantification of IL-6. Experiments were performed as described in the manufacturer’s protocol (human IL-6, from BD, OptEIA, Heidelberg, Germany).

### Cell viability assay

After collecting the supernatants of treated RASFs, cells were incubated with CellTiter-Blue reagent following the instruction of the manufacturer (G8081, Promega). By determining the reduction from resazurin to resorufin, the cell viability was estimated and quantified to reflect the toxic effect of each treatment.

### Statistics

All data were presented from at least three independent experiments. GraphPad Prism (GraphPad software Inc., California, USA) was used for data analysis. The statistic tests used are given in the figure legends. When data are presented as line plots, the line represents the mean. When data are presented as bar charts, the top of the bar represents the mean and error bars depict the standard error of the mean (SEM). When data are presented as box plots, the boxes represent the 25th to 75th percentiles, the lines within the boxes represent the median, and the lines outside the boxes represent the 10th and 90th percentiles. The level of significance was *p* < 0.05.

## Supplementary Information

**Additional file 1: Supplement fig. 1.** Original and uncropped images of WB.

## Data Availability

The datasets used and/or analyzed during the current study are available from the corresponding author on reasonable request.

## Abbreviations

BZD: Benserazide hydrochloride
CLG: Clorgyline
COMT: Catechol-O-methyltransferase
CRP: C-reactive protein
DA: Dopamine
DAT: Dopamine transporter
DBH: Dopamine-beta-hydroxylase
DDC: DOPA decarboxylase
ELISA: Enzyme-linked immunoSorbent assay
EPI: Epinephrine
GABA: γ-aminobutyric acid
IDA: Indatraline hydrochloride
IF: Immunofluorescence
IL: Interleukin
JNK: Jun activated kinase
LB: Lazabemide hydrochloride
MAO: Monoamine oxidase
NE: Norepinephrine
NET: Norepinephrine transporter
NFκB: Nuclear factor ‘kappa-light-chain-enhancer’ of activated B-cells
NSS: Normal swine serum
OA: Osteoarthritis
PNMT: Phenylethanolamine N-methyltransferase
qRT-PCR: Quantitative real-time polymerase chain reaction
RA: Rheumatoid arthritis
ROS: Reactive oxygen species
RSP: Reserpine
SEM: Standard error of mean
SF: Synovial fibroblast/s
SNS: Sympathetic nervous system
TH: Tyrosine hydroxylase
TNF: Tumor necrosis factor
VMAT: Vesicular monoamine transporters
WB: Western blotting
