# Supplementary material for: Components of the sympathetic nervous system as targets to modulate inflammation – rheumatoid arthritis synovial fibroblasts as neuron-like cells?
Source: J Inflamm (Lond). 2023 Mar 14;20:9. doi: 10.1186/s12950-023-00336-z (PMC10015726; doi:10.1186/s12950-023-00336-z)
Supplement: Supplementary file 1 — Additional file 1: Supplement fig. 1. Original and uncropped images of WB. [file 12950_2023_336_MOESM1_ESM.docx]

**Supplement figure 1**


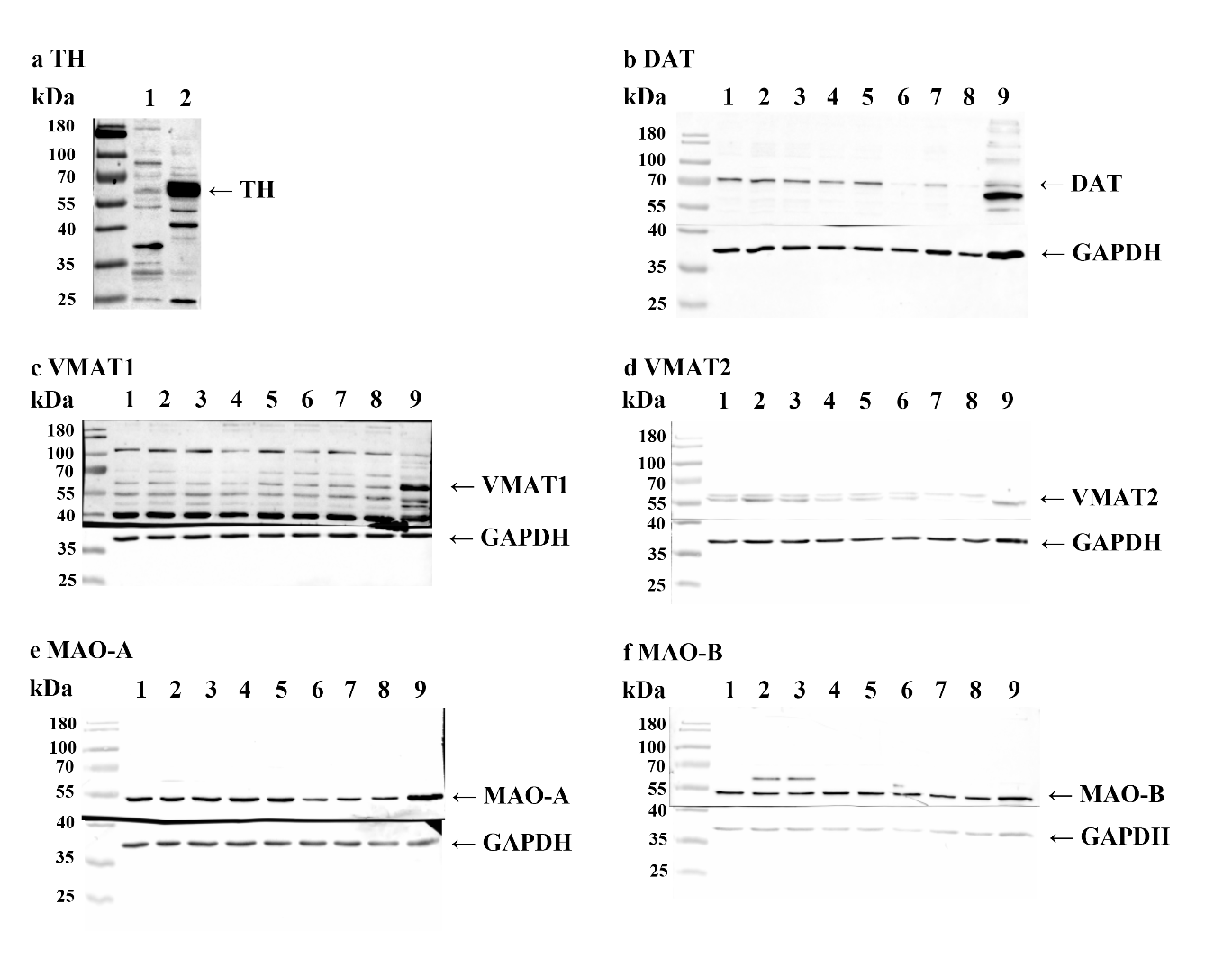


**Supplement figure 1 Original and uncropped images of WB.**

Original and uncropped WB image of TH (Column 1, RASFs; Column 2, mouse brain protein as a positive control) **(a),** DAT **(b),** VMAT1 **(c),** VMAT2 **(d),** MAO-A **(e)** and MAO-B **(f)**. **b-f)** Columns 1-8, RASFs under different stimulations; Column 9, mouse brain protein as a positive control.
